# Mapping the Worldwide Trends on Energy Poverty Research: A Bibliometric Analysis (1999–2019)

**DOI:** 10.3390/ijerph18041764

**Published:** 2021-02-11

**Authors:** Yiming Xiao, Han Wu, Guohua Wang, Hong Mei

**Affiliations:** College of Public Administration, Huazhong University of Science and Technology, Wuhan 430074, China; xiaoehust@163.com (Y.X.); mpawin@hust.edu.cn (G.W.); meihong_26@163.com (H.M.)

**Keywords:** bibliometrics, energy poverty, energy consumption, social network analysis

## Abstract

Energy poverty is one of the main challenges facing humanity in the 21st century. Research on energy poverty is becoming a common focus of scholars in many areas. Bibliometrics can help researchers dig deep into the information of specific research fields from a quantitative perspective. In this study, we collected 1018 research papers in the field of energy poverty published in the period 1999–2019 from the Web of Science databases and conducted a bibliometric analysis on them. Cleaning and screening of sample papers, matrix construction, and visualization were performed using Bibliometrix, VOSviewer, and HistCite, summarizing the internal and external characteristics of the papers. With regard to external characteristics, a total of 982 research institutions in 80 regions conducted research in this field. There is extensive cooperation between the countries, and the UK, the USA, Australia, and Italy play the most active role in the cooperation network. With regard to internal characteristics, we found the two most representative citation paths: one path starts from the concerns of energy-poor groups and stops at an ethical discussion on energy poverty; the second path is based on the existing technological path, continuously developing coping policies, evaluation methods, and a conceptual framework for dealing with energy poverty. Furthermore, through coupling analysis, we discovered four focuses of energy poverty research: improvement of definition, improvement of evaluation methods, effects of coping policy, and energy justice. Through a comprehensive analysis of existing papers, this paper reveals some limitations of previous studies and recommends some promising directions for future research on energy poverty.

## 1. Introduction

The history of human society is closely linked to the evolution of energy utilization [1]. Energy is one of the most basic conditions needed to sustain people’s daily lives [2]. “Energy poverty” refers to a situation in which it is difficult for people to obtain adequate, affordable, high-quality, safe, and environmentally friendly energy services to live a decent life [3]. Energy poverty, which is widespread in all regions of the world, is becoming a great challenge for humanity, which wants to achieve the New Millennium Development Goals [4]. In Europe, there are about 50 million to 125 million people living in energy poverty, and the size of this group has been increasing in recent years [5]. In the underdeveloped regions of Asia and Africa, more than half of the total population has to live in energy poverty [6,7]. According to the prediction of the International Energy Agency, if effective measures are not taken to mitigate energy poverty, there will still be about 2.5 billion people in the world who will not have access to clean and reliable energy by 2030 and can only rely on traditional biomass energy as their daily energy source [8]. Energy poverty not only affects health and diminishes happiness of residents [9,10] but also hinders the sustainable development of economy and environment [11]. On 9 November 2011, the United Nations designated 2012 as the International Year of Sustainable Energy for All and set the popularization of modern energy services as one of the important energy goals for 2030 [12].

Boardman [13] first gave a quantitative definition of energy poverty from an energy consumption perspective: families that cannot get enough energy services at a cost of less than 10% of their income are in a state of energy poverty. Some scholars have tried to incorporate energy efficiency and environmental impact in energy poverty research and have constructed a more complete conceptual framework for energy poverty [14,15,16]. In response to appeals from scholars and the public, European countries, especially the United Kingdom (UK), France, and Cyprus, began to address the issue of energy poverty within their borders many years ago and tried to formulate appropriate policies to alleviate energy poverty [17]. India, China, and other developing countries have gradually begun to include energy poverty in their policy agendas, but have achieved only limited effects of policy works [18,19]. Following the outbreak of the COVID-19 pandemic, lockdown measures taken by almost all countries and declining income of residents further exacerbated the already severe situation of energy poverty [20,21]. Currently, the grim reality of energy poverty requires a more in-depth research of its causes, consequences, and solutions.

In the last 20 years, a lot of research results have been accumulated in the field of energy poverty. Especially after 2015, the number of related research papers increased rapidly at a rate of more than 100 papers per year. To better understand the current situation in this field, some scholars have reviewed existing papers from different perspectives, focusing on the following four areas: (1) Definition: Castaño [17] reviewed related definitions of energy poverty, while Day [1] presented a more comprehensive and coherent conceptual framework based on previous studies; (2) Evaluation method: Nussbaumer [22] and Herrero [23] reviewed existing energy poverty evaluation indicators and summarized the advantages and disadvantages of these indicators; (3) Consequences: Sovacool [4] and Jessel [24] integrated existing research data and discussed health hazards, environmental pollution and gender discrimination caused by energy poverty; and (4) Coping policies: Bouzarovski [25] systematically collected and evaluated policies being implemented to address energy poverty in various countries. These literature reviews provide an overview of past research results, allowing researchers and government to better understand the practical condition so that they can better plan their research projects and formulate relevant policies [26]. However, the authors of these reviews generally chose a limited number of papers from renowned journals for review based on their experience, so they would inevitably miss some important papers when doing paper collection. Therefore they lack a real international perspective. For this reason, existing reviews barely reflect the overall research on energy poverty [27].

Bibliometrics provide quantitative methods for analyzing academic literature in specific research fields. Using bibliometrics methodology, researchers can conduct a quantitative analysis of the distribution structure and evolution of disciplines in papers, thus minimizing the impact of subjectivity on the quality of review [28,29]. In the field of energy research, some scholars have applied the bibliometric method to energy security, energy efficiency, the renewable energy supply chain, and other fields [30,31,32]. According to data from the Web of Science (WoS), more than 500 papers published from 2015 to 2019 directly relate to energy poverty, but there were no bibliometric studies of energy poverty covering the entire period. To get a clearer picture of the research on energy poverty and to explore the directions for future development, it is necessary to perform a comprehensive bibliometric study.

The characteristics of papers can generally be divided into external and internal. The external characteristics include publication time, country, institution, author, journal, etc., while the internal characteristics include keywords, research focus, and references. To ensure the comprehensiveness of the research, bibliometrics research should start from the internal and external characteristics of papers. In this study, we performed a quantitative analysis of the internal and external characteristics of papers focused on energy poverty (published from 1999 to 2019) using bibliometrics and its supporting visualization methods, to understand the commonalities and differences of previous studies in the field of energy poverty.

To comprehensively summarize the existing energy poverty studies and provide valuable insights into the practice of government policies, we try to explore the answers to the following two sets of questions:

With regard to external characteristics, which countries, institutions, journals, and authors are most influential in energy poverty research? What is the cooperative relationship between different entities?

With regard to internal characteristics, what are the focuses of energy poverty research? What are the development paths that energy poverty research has traversed? Are there any unexplored areas in this field?

The rest of this paper is organized as follows: The second part describes the data sources and research methods used in this study. The third part presents a visualization of the results of bibliometric analysis and discusses the results of the analysis. The fourth part provides the conclusions of this study and presents some suggestions for future research on energy poverty.

## 2. Methodology

**Figure 1:**
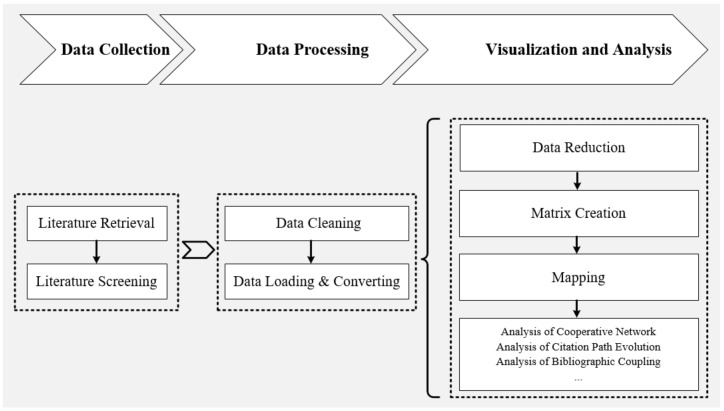
Research framework of bibliometric analysis.

Bibliometrics is a technology used to investigate the development process and knowledge structure of a particular field of research [33]. Bibliometrics can be used to depict the overall picture of a particular research field at the macro level, and it can also be used to analyze hot topics at the micro level, so more and more scholars have applied it in their research [34,35]. With the support of mathematics and statistics, bibliometrics can be applied to the analysis of various literature samples such as books, periodicals, and policy texts [36,37]. The standard bibliometrics procedure consists of the steps of document collection, data processing, visualization, and analysis. Relying on the research of Li [27], Zupic [38], Aria [39], and other scholars in bibliometrics, we formulated our own research framework shown in Figure 1.

### 2.1. Data Sources

Selection of databases: Compared to Google Scholar, PubMed, Springer, Wiley, and other databases, the Web of Science Core Collection includes more reputable journals and related papers. This database has a strict literature screening mechanism, which ensures a high academic level of collected papers [40]. Standardized literature entries exported from the Web of Science Core Collection can be directly applied in various bibliometric analysis tools. Considering the comprehensiveness and quality of the collected papers, most scholars rely on the Web of Science Core Collection to conduct a bibliometric study [41,42]. In this study, we collected relevant research papers with a complete research structure from four databases in the Web of Science Core Collection, namely Science Citation Index Expanded (SCI-Expanded), Social Sciences Citation Index (SSCI), Arts & Humanities Citation Index (A&HCI), and Emerging Sources Citation Index (ESCI).

Selection of keywords: There are multiple expressions of energy poverty, among which “energy poverty,” “energy poor,” and “fuel poor” are often used interchangeably [16,43]. There are no obvious differences between these concepts; only the referred regions or climatic conditions differ [44]. Therefore, this study follows the practice of previous studies, i.e., we used the keywords “energy poverty,” “energy poor,” “fuel poor,” and “fuel poverty” to search papers [45].

Defining the retrieval period: In the selected databases, the first paper related to energy poverty appeared in 1999, and the number of related studies has been increasing since then. In this study, the retrieval period is set to 1999–2019.

#### 2.1.1. Methods of Retrieval

We constructed the following search strings:

#1: TS = “energy poverty” OR TS = “energy poor” OR TS = “fuel poverty” OR TS = “fuel poor”

#2: TI = (“energy poverty” OR “energy poor” OR “fuel poverty” OR “fuel poor”) OR AK = (“energy poverty” OR “energy poor” OR “fuel poverty” OR “fuel poor”) OR KP = (“energy poverty” OR “energy poor” OR “fuel poverty” OR “fuel poor”)

#3: #1 NOT #2

The rules for retrieval in Web of Science are as follows: TI means “title”; AK means “author keywords”; KP means “KeyWords Plus”; TS means “topic,” covering the following four fields: “title,” “author keywords,” “KeyWords Plus,” and “abstract”; “A AND B” means “Find all records that appear in both set A and set B,” while “A NOT B” means “Find all records in set A that are not in set B.”

#### 2.1.2. Methods of Screening

**Figure 2:**
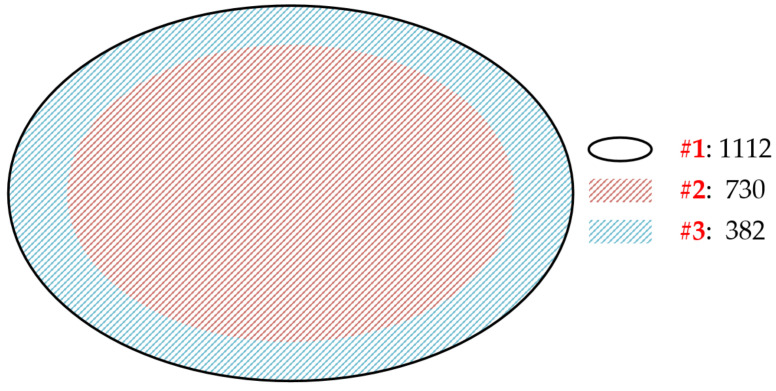
The inclusion relations between #1, #2, and #3.

The search string “#1” returns those studies that match the search terms in titles, author keywords, KeyWords Plus, or abstracts, totaling 1112. The search string “#2” returns those studies that match the search terms in titles, author keywords, or KeyWords Plus, totaling 730. The search string “#3” returns those studies that match the search terms in abstracts but not in titles, author keywords, or KeyWords Plus, totaling 382. As shown in Figure 2, “#2” and “#3” constitute the entirety of “#1.”

The 730 documents (returned by “#2”) are highly relevant to energy poverty, because energy poverty occurs in the titles, author keywords, or KeyWords Plus. These documents can be directly included in the study without screening.

On the contrary, we need to screen the 382 documents (returned by “#3”) since energy poverty only occurs in the abstract but not in titles or keywords. After excluding irrelevant documents, 296 documents relevant to energy poverty are obtained.

In total, 1026 documents (730 + 296) are imported into Bibliometrix for processing, and 8 documents are removed because of lack of information. Finally, we obtain 1018 documents for analysis. The retrieval and screening was conducted on 27 October 2020.

### 2.2. Analysis Methods

In this study, the Bibliometrix3.0.3 Package (Download from: bibliometrix.org) based on the R language was used to process the collected papers. Bibliometrix is an open source tool developed by Massimo Aria et al., which is used for data processing as well as for scientific knowledge mapping and analysis. It can be used to easily convert data exported from WoS databases into the R data frame, on the basis of which data can be cleaned, filtered, and analyzed, and the matrix construction can be performed. Currently, this tool is used in bibliometric studies by scholars in many fields [39]. VOSviewer (Download from: vosviewer.com), developed by Nees Jan van Eck and Ludo Waltman, is a bibliometric tool mostly used to construct and visualize co-occurrence relationships in the literature [46]. HistCite (Download from: clarivate.com), developed by Eugene Garfield, is a bibliometric tool mostly used to translate citation relationships into citation paths. VOSviewer1.6.15 and HistCite12.03.17 were also used to conduct more explanatory visual analysis of relevant data.

## 3. Results and Discussion

### 3.1. External Characteristics of the Publications

#### 3.1.1. Overall Status of the Publications

**Figure 3:**
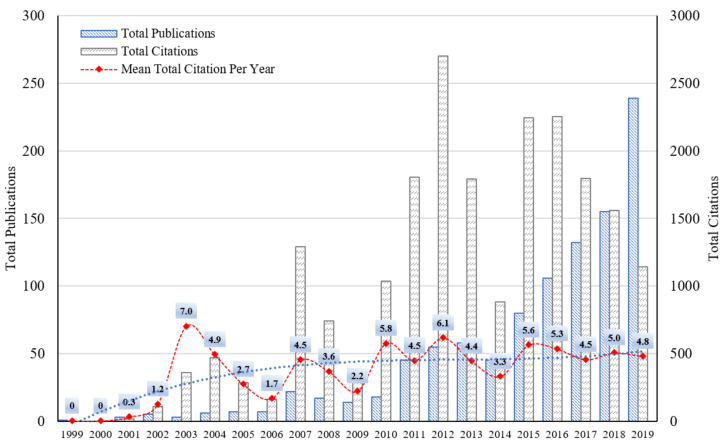
Annual distribution of total publications (TP), total citations (TC), and the mean total citation per year (MTCY).

Figure 3 shows the overall trend of publications in energy poverty in the period 1999–2019. Blue and gray bars represent the annual number of total publications (TP) and total citations (TC), respectively. The red dots represent the mean total citation per year (MTCY).

The annual number of TPs exhibited an overall increasing trend in the period 1999–2019. Following the practice of Xie [47], we segmented the entire period into three stages according to the annual publication amount, i.e., 1999–2010 (annual publications were below or around 20), 2011–2015 (annual publications were between 20 and 100), and 2016–2019 (annual publications were more than 100). Prior to 2010, the annual number of TPs on energy poverty was less than 20 per year, and sometimes it was zero. This shows that energy poverty, a completely new concept, has not attracted much attention from scholars. Research on energy poverty was in its initial stage in this period. During 2011–2015, the annual number of TP on energy poverty increased at a rate of 57 papers per year, indicating that energy poverty was gradually becoming one of the important topics in the field of energy research, and research on energy poverty entered a growing stage. After 2016, energy poverty research entered an active period with high yield. The annual number of TP on energy poverty was increasing at a rate of 100 papers per year and reached the maximum number of 239 in 2019.

Both TC and MTCY show yearly fluctuations. In the early research stage, the concept of energy poverty and the research methodology were not mature. So, there were no high-quality or high-influence papers in some years. This explains the low TC and the drastic fluctuation of MTCY in the early papers. In 2003, MTCY reached the highest value of seven, which means that the papers published in that year have been cited by seven papers each year since then. This shows that the papers published in 2003 have a high influence, laying the foundations for the field of energy poverty. After 20 years of development, MTCY exhibited a steady development trend in 2018 and 2019, indicating that research on energy poverty has entered the maturity stage. Supported by a complete set of concepts and methodologies, scholars carried out research in a more or less predictable manner, which also had a limiting effect on the groundbreaking research.

#### 3.1.2. Contributions by Countries

The output of energy poverty research is counted from a national perspective. Papers authored by scientists from multiple countries are counted in the output of all relevant countries. Following this approach, we can figure out the full output of one country and the cooperation among countries.

**Figure 4:**
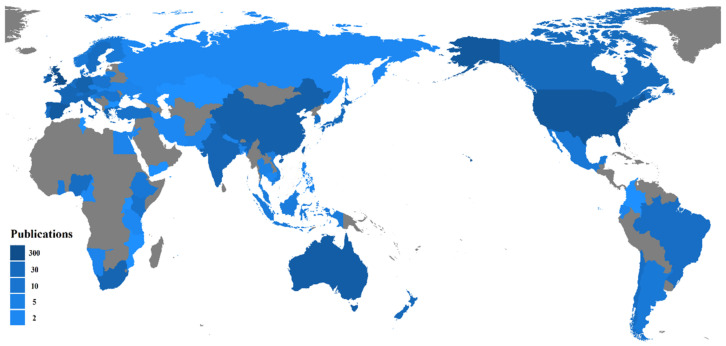
Production distribution in the field of energy poverty.

From 1999 to 2019, scholars from 80 countries or regions published papers related to energy poverty. Table 1 shows the number of published papers, the proportion of published papers, the number of citations, and the h-index of the top 20 most productive countries or regions. The data map in Figure 4 shows the numbers of published papers contributed by all countries or regions in the world. With 335 published papers, the UK is the country with the highest number of published papers. The UK and the USA account for almost half of the total number of publications, far exceeding other countries or regions combined. Governments in developed countries can provide more resources and data support to scholars to conduct research on energy poverty. That is why all five top countries are developed countries. With the development of the economy and the strengthening of civic awareness, scholars from developing countries, including China, South Africa, India, and Nigeria, have begun to analyze the phenomenon of energy poverty in their countries and they have been the most active in the field of energy poverty in recent years.

The h-index is usually defined as the following expression: A scientist has an index h if the h of his/her papers has at least h citations each, and other papers are cited no more than h times each [48]. At present, the h-index is widely used to assess the influence of publications produced by countries, institutions, scholars, and even the influence of papers published in certain years [31]. The total citations and h-index of the UK and the USA are far higher than those of other countries. The h-index of most countries is basically between 10 and 20, except the UK and the USA. China, which ranks 6th in the number of published papers, has an h-index of 12, while Denmark, which ranks 20th in the number of published papers, has an h-index of 10. This indicates that there is no positive correlation between the quantity and quality of publications. Countries with a small share of literature contribution in the total number of publications can also produce research results that are generally recognized in academic circles.

**Figure 5:**
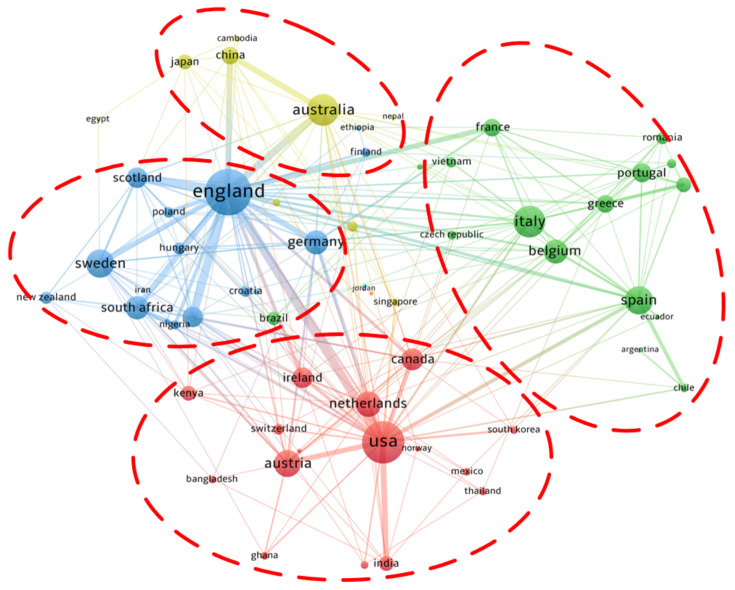
Cooperation network between countries in the field of energy poverty.

Figure 5 shows the cooperation relationship between countries or regions. The size of the node indicates the importance of a country in the whole network. The thickness of the line indicates the intensity of cooperation between two countries. Figure 5 shows that the cooperation network of countries can be divided into four clusters, and the UK, the USA, Australia, and Italy are at the core of each country cluster. The UK has established cooperative relations with almost all important regions and is the leader of the whole cooperative network. Most countries tend to cooperate with other countries in the same region. Due to similarities in geographical conditions, economy, and even culture, countries in the same region can use similar research paradigms, making it easier for them to establish cooperative relations.

#### 3.1.3. Contributions by Institutes

A total of 982 research institutions have published papers related to energy poverty. As shown in Table 2, The University of Manchester in the UK and Columbia University in the USA occupy the first two places in the ranking, with 28 and 22 papers, respectively. Among institutions, 11% of them published two papers, and 65% of them published only one paper. This indicates that energy poverty research is scattered across institutions. At the institutional level, there is no agglomeration trend similar to Bradford’s law or Lotka’s law.

**Figure 6:**
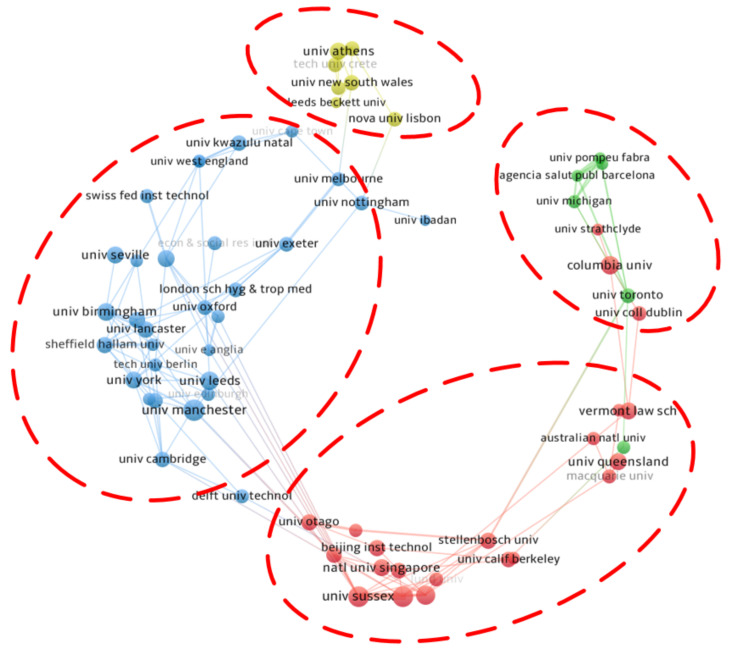
Cooperation network between institutions in the field of energy poverty.

Compared to the international cooperation network, the density of the institutional cooperation network is lower, which is characterized by more clusters. Figure 6 shows that among the four institutional clusters there is only bilateral cooperation, and there has been no cooperation spanning across three clusters. The University of Sussex is most active in collaborating with other institutions, but most of its partners are institutions in the same country. Similar to the University of Sussex, most institutions also tend to establish cooperative relations with other domestic research institutions. The most representative are the cooperation groups led by the University of New South Wales, the University of Michigan, and the University of Birmingham. In the institutional cooperation network, a few nodes, such as the University of Nottingham, the University of Cape Town, the University of Toronto, and so on, play the role of “bridge,” connecting cooperation groups in different regions.

#### 3.1.4. Distribution of Journals

**Figure 7:**
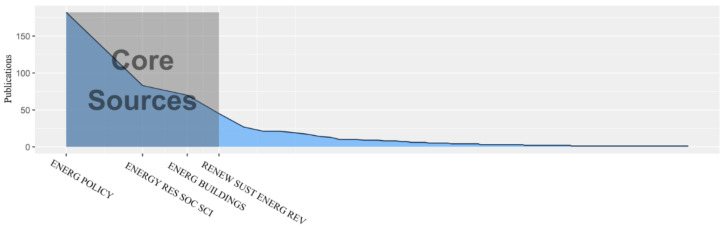
Publication amount of journals in the field of energy poverty.

A total of 283 journals published papers related to energy poverty in the study period. Among the 20 most productive journals, *Renewable and Sustainable Energy Reviews* is the one with the highest impact factor, representing the highest level of energy poverty research to some extent. *Energy Policy*, *Energy Research and Social Science*, *Energy Building*, and *Renewable and Sustainable Energy Reviews* are the top four most productive journals by the number of published papers. As shown in Figure 7, the above four journals account for 37% of all published papers related to energy poverty, forming an essential part of energy poverty research, confirming Bradford’s law. Simply put, Bradford’s law is the embodiment of the Pareto effect with the following quantitative characterization: if journals in one field are divided into three parts by the number of papers, each part accounting for about one-third of all papers, then the journals in each part will be proportional to 1:n:n^2^, while the first part is usually defined as the core part.

**Figure 8:**
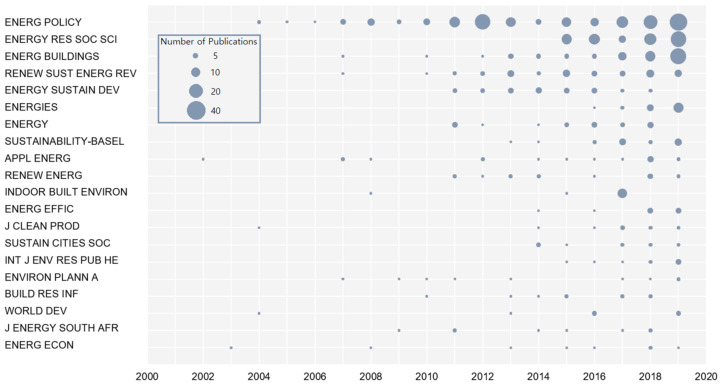
The top 20 most productive journals in the field of energy poverty.

Figure 8 shows the annual variations in the number of papers published in the top 20 journals. *Applied Energy*, which has been publishing papers on energy poverty since 2002, is one of the reputable journals that initially paid attention to this field. Since 2011, more journals have begun to pay attention to energy poverty, and the number of publications has been steadily increasing. In the last 10 years, *Energy Policy* has paid attention to energy poverty. With 182 published papers, it became the main contributor to the increase of literature in the field, boasting the largest number of citations. It is worth noting that journals that publish energy poverty studies cover a wide range of disciplines, including environmental science, management, economics, architecture, etc., reflecting the interdisciplinary nature of energy poverty research.

#### 3.1.5. Contributions by Authors

**Figure 9:**
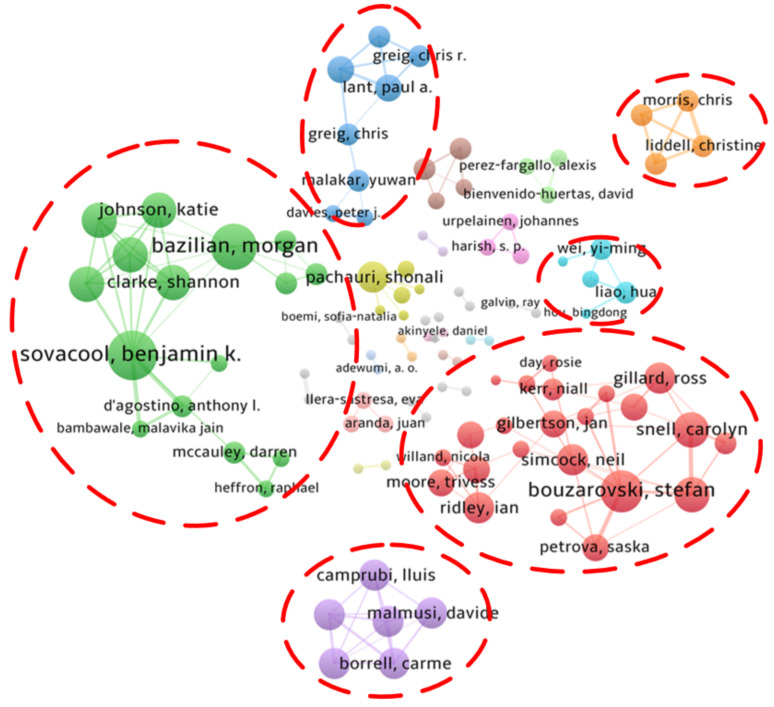
Cooperation network between authors in the field of energy poverty.

A total of 2253 authors have published papers in the field of energy poverty. Among published papers, 210 were written by single authors, while others were co-written by several authors. In general, each paper was completed by an average of three authors. Compared to international cooperation networks and institutional cooperation networks, the co-authorship networks shown in Figure 9 lack global connections. Apart from authors in the large-scale co-authorship networks represented by Sovacool and Bouzarovski, most authors exist in networks in the form of small co-authorship networks or isolated nodes. This shows that current academic cooperation between scholars dealing with energy poverty is inadequate and that the scholars do not fully share relevant knowledge and opinions.

**Figure 10:**
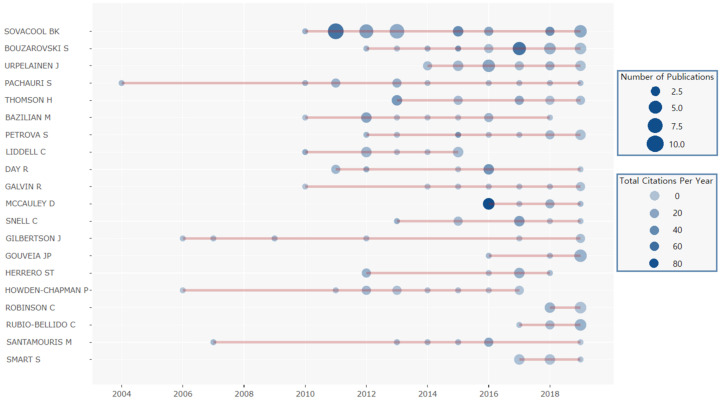
The top 20 productive authors in the field of energy poverty.

Figure 10 shows that among the top 20 most productive scholars, Pachauri published his first paper on energy poverty in 2004 and has maintained a stable output ever since. Sovacool started publishing relevant papers relatively late in 2010, but he published nine papers in the next year and gained a large number of citations. For most scholars, once they started the research on energy poverty, they would continuously publish papers, which indicates the high research significance of energy poverty. The papers published by Sovacool in 2011, McCauley in 2016, and Bouzarovski in 2017 have the most numerous citations, which indicates that these publications have played an important role in the history of energy poverty research. In the latter part of the analysis, we will conduct a more in-depth discussion of these important papers.

### 3.2. Internal Characteristics of Publications

This section discusses in detail the internal characteristics of papers related to energy poverty to shed light on development paths and research focus in this field. Keywords can effectively reflect the focuses of the study [47]. Thus, the primary task is to analyze the frequencies and evolution of keywords. Firstly, keywords with similar meanings such as “housing” and “household,” “poverty” and “deprivation” are merged. Then, the words that are used in paper retrieval or have a broad meaning, such as “energy poverty,” “fuel poverty,” “energy,” “policy,” and so on, are deleted. Finally, visualization tools are used to draw a tree map that reflects the frequencies of keywords, and a Sankey map that reflects the evolution of keywords.

**Figure 11:**
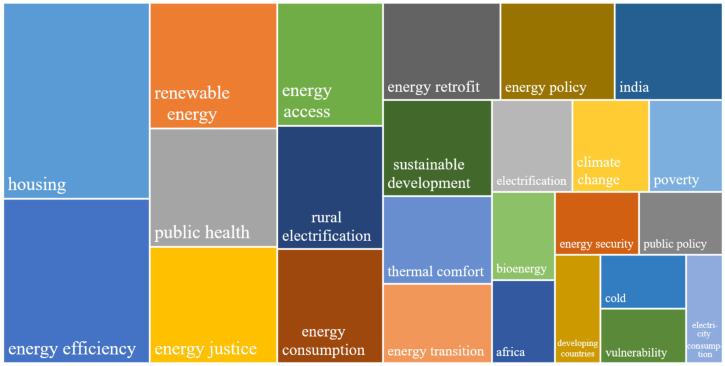
Tree map of high-frequency keywords in the field of energy poverty.

The tree map shown in Figure 11 contains words in the fields of energy, economy, health, construction, and even policy, indicating that energy poverty is an interdisciplinary topic. The word “housing” appears in nearly 10% of papers, which indicates that the main research object of energy poverty is residential houses. Furthermore, “energy efficiency” and “renewable energy” appear in 8% and 5% of the papers, respectively, which shows that scholars consider the improvement of energy efficiency and the use of renewable energy as two important solutions to energy poverty. The appearance of “public health” and “thermal comfort” indicates that the ultimate goal of energy poverty research is to improve the health and ensure well-being of the population. In addition, the fact that the word “energy justice” appears in 5% of papers indicates that some researchers in the field of energy poverty have drawn attention to ethical aspect of the problem.

**Figure 12:**
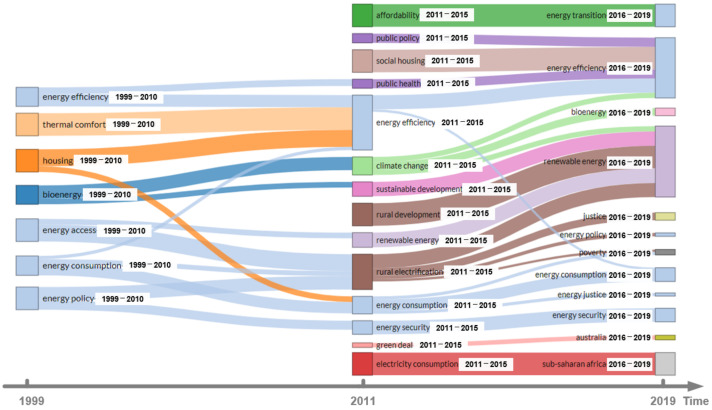
Keywords’ evolution in the energy poverty research.

The Sankey map in Figure 12 shows the evolution pattern of keywords. In the history of energy poverty research, the evolution of keywords has been quite volatile. Prior to 2010, keywords in the literature were relatively concentrated, and scholars discussed the basic problems of energy poverty from the perspective of energy consumption, energy policy, housing and thermal comfort. After 2011, these keywords underwent an obvious fusion and regeneration: “energy acquisition,” “energy consumption,” and “energy policy” first evolved to “rural electrification,” and then new keywords such as “energy justice” appeared. After 2011, “biomass energy” was replaced by “climate change” and “sustainable development” and converted to “renewable energy”; the keywords “household” and “thermal comfort” evolved into energy efficiency research and then lost their dominant position; the keywords “acceptability,” “social housing,” and “green trading” first appeared after 2011 and changed after 2016. In general, the number of keywords in the field of energy poverty is constantly increasing, and keywords cover quite a few research fields, indicating that the interdisciplinary nature of energy poverty research is becoming more and more notable. In the following citation path analysis and coupling structure analysis, we explore the general law of literature evolution from a microscopic perspective.

#### 3.2.1. Analysis of Citation Path Evolution

The citation relationship between papers reveals the paths of knowledge dissemination. In the initial stage of research in a specific field, the citation relationship is relatively simple. As the research breadth expands, the citation relationship between papers will gradually evolve into a vast citation network [49]. The citation analysis was first proposed by Hummon [50]. At present, the analysis of citation path evolution has been adopted for bibliometrics research in various fields [51]. By analyzing citation relationships and extracting key citation paths, researchers can discover the development process of important papers from a microscopic perspective [36].

**Figure 13:**
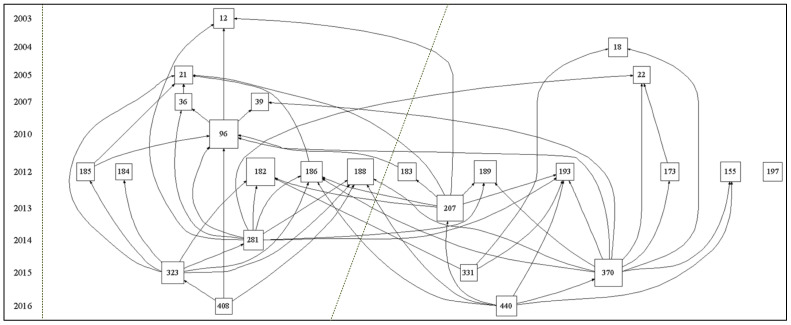
Overall citation paths of energy poverty research from 1999 to 2019.

**Figure 14:**
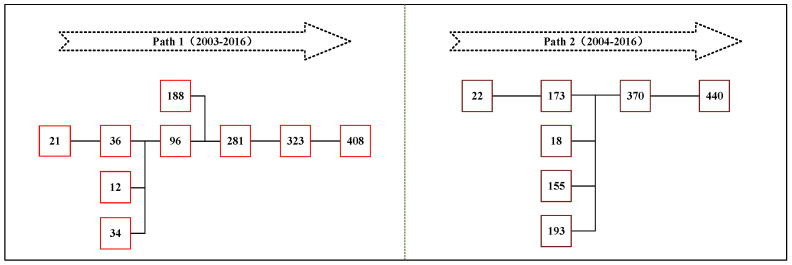
Key citation paths of energy poverty research.

In this study, paper samples were sorted according to the number of citations, and citation paths of the 25 most cited papers were visualized using HistCite software. Figure 13 shows that the network begins with papers #12 [52] and #18 [53] and ends with papers #408 [54] and #440 [1]. Except for paper #197 [55] which is an isolated point, other papers entered the citation network from 2003 to 2016. The number of papers published in 2012 is higher than in any other year, which indicates that the research in that year has an important impact on the diffusion of knowledge throughout the field. Based on the network density and the importance of citations, the whole network can be divided into left and right parts. Therefore, the two most representative citation paths were identified as shown in Figure 14.

The first path originates from the discussion of the living conditions of people suffering from energy poverty and settles on the ethical concept of energy justice. Paper #12 [52] analyzes the causes of an abnormally high number of deaths in 14 European Union countries in winter and points out that inadequate heating of residential houses can significantly increase the mortality rate. This paper, for the first time, systematically discusses the negative effects of energy poverty on residents and calls on the government to pay more attention to energy-poor groups. Paper #21 [56] and #36 [57] analyze the impact of energy poverty on quality of life, physical health, and even mental health, based on data obtained from in-depth interviews with families in various communities. Going further in the direction of the above studies, the authors of paper #96 [58] conducted a more comprehensive analysis of the potential impact of energy poverty on human health based on a large-scale survey of different groups (adults, teenagers, and children) lasting 10 years, emphasizing that children are one of the main victims of energy poverty. The authors of paper #188 [59] noticed the inequality suffered by several underprivileged groups as a result of energy poverty and suggested for the first time that energy poverty is linked to different concepts of social and environmental justice. Based on previous studies, papers #281 [60] and #323 [61] reflect the causes of energy poverty in different regions at the microscopic level. Finally, paper #408 [54] applies the principle of justice to the energy system and conducts a detailed discussion of the emerging ethical concept of energy justice.

The second path concerns the development the coping policies, evaluation methods, and a conceptual framework related to energy poverty, with the aim of effectively measuring and solving the problem of energy poverty. Paper #22 [62] analyzes the contradiction between energy poverty and the goal of the United Nations Framework Convention on Climate Change and proposes the elimination of energy poverty by levying incremental tax on oil and establishing an “energy poverty alleviation fund.” Paper #173 [4] explains the correlation between energy availability and the Millennium Development Goals, pointing out that energy poverty has a major negative impact on society, environment, and even global issues such as climate warming. Papers #18 [53], #155 [22], and #193 [63] examine existing methods for assessing energy poverty and strategies for formulating poverty reduction policies and set out reasonable procedures for assessing energy poverty and formulating countermeasures. Based on the above studies, paper #370 [64] provides a comprehensive conceptual framework for studying and mitigating energy poverty in developed and developing countries. Paper #440 [1] further expands the original conceptual framework of energy poverty from a capability perspective, so that the framework can be used to comprehensively measure energy poverty in different regions of the world.

#### 3.2.2. Analysis of Bibliographic Coupling

**Figure 15:**
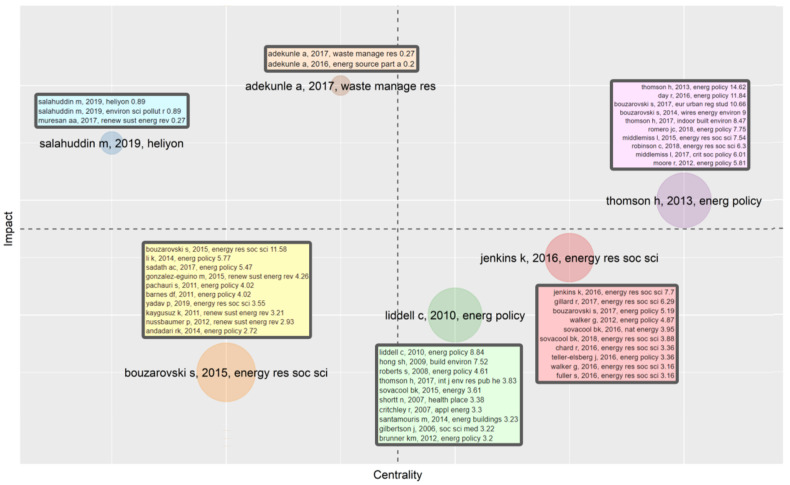
Clustering of bibliographic coupling in energy poverty research.

Bibliographic coupling analysis is a common method for establishing network and clusters of literatures [65,66]. It can integrate papers throughout the whole period and explain the focus of research in a given field [67,68]. According to bibliographic coupling analysis, we established the coordinate diagram, as shown in Figure 15 [39]. After reviewing representative papers, we grouped them into the following six clusters.

##### Cluster 1: Improvement of the Definition of Energy Poverty

Cluster 1, represented by Thomson [69], revised and expanded the original definition of energy poverty. Moore [70], Middlemiss [71], and Robinson [72] criticized several definitions adopted by the British government. Robinson analyzed the UK’s energy poverty situations based on “10% definition” and “LIHC definition,” pointing out that neither of them can accurately reflect the complex spatial distribution of energy poverty. Moore analyzed the deviation of previous definitions and pointed out that the future definitions should take into account practical factors such as family size and energy efficiency. Finally, to make the definition operational for different environments, Day [1] proposed a broader definition based on “capacity theory.” Improving the definition not only promotes the development of energy poverty research but also helps the government to identify energy-poor groups and formulate coping policies.

##### Cluster 2: Improvement of Energy Poverty Evaluation Methods

Cluster 2, represented by Bouzarovski [64], pays more attention to the improvement of energy poverty evaluation methods. Pachauri [73] suggested formulating different evaluation methods at the macro, community, and household levels. Bouzarovski [64] pointed out that energy poverty is essentially the ineffective operation of socio-technical paths. Therefore, when measuring energy poverty, researchers should consider the composition of energy services provided to households. Based on previous studies, Nussbaumer [22] proposed a new index named the multidimensional energy poverty index (MEPI) and conducted a comprehensive analysis of energy poverty in African countries. Kaygusuz [74], Barnes [75], and Sadath [76] studied energy poverty in rural areas of developing countries. Barnes found that more than half of rural households in Bangladesh had some difficulty in obtaining energy. Sadath’s research in India confirmed that the existence of energy poverty often coincides with other forms of deprivations such as income poverty. Given that women play an important role in home cooking and heating, both Kaygusuz and Sadath believed that women face gender inequality in energy poverty situations. In summary, through the improvement and application of energy poverty evaluation, relevant studies have enriched the methodology for measuring energy poverty, which allows the government to better understand the state of energy poverty and thus pay more attention to vulnerable groups in energy use.

##### Cluster 3: Effects of Coping Policies

Cluster 3, represented by Liddell [58], seeks to analyze the effects of different policies that address energy poverty. The Warm Front Scheme implemented by the UK government has interested many scholars. Gilbertson [77] conducted semi-structured interviews with some families benefiting from the project and found that the project not only improved the physical health but also improved family relations and enhanced the emotional security of family members. A large-scale field survey conducted by Hong [78] also confirmed Gilbertson’s research results. After investigating the costs and benefits, Sovacool [79] found that the project brought energy consumption savings and annual income growth to residents. However, according to survey conducted by Critchley [57], the Warm Front Scheme failed to achieve the expected results for old houses, and families living in those houses may face a higher risk of anxiety or depression due to inadequate heating. Roberts [80], Santamouris [81], and Thomson [9] also discussed the adverse consequences caused by the lack of relevant policies. Overall, European countries are the first regions to implement policies to address energy poverty and the effects of their policies have been fully studied. By contrast, few researchers have paid attention to relevant policies in developing countries, although there may be more room for improvement in their policy practices.

##### Cluster 4: Ethical Discussion on Energy Poverty: Energy Justice

Cluster 4, represented by Jenkins [54], reflects the recent ethical discussion on energy poverty. Scholars pointed out that access to energy services should be a basic right of citizens in modern society, and research in the field of energy should not be conducted in a “moral vacuum” [82]. Based on this understanding, Walker [59] defined energy poverty as a type of injustice and decomposed the injustice into three dimensions: procedure, distribution, and cognition. Sovacool [82] built an energy justice framework focused on availability, affordability, due process, transparency and accountability, sustainability, equity, and responsibility. Walker [83] emphasized the dynamic characteristic of energy justice and proposed to include the public deliberations about necessities in the scope of justice. Based on previous studies, Jenkins [54] outlined the evaluative and normative reach of energy justice to help future scholars identify the regions where energy injustice occurs. Furthermore, Gillard [84] and Bouzarovski [85] emphasized the difference between region-centric energy justice and people-centric energy justice.

##### Cluster 5 and Cluster 6: Technology Strategy and Carbon Emission Effect

There are two more clusters with only a small number of papers. Cluster 5 discusses the possibility of using biomass resources for fuel cell production in energy-deficient areas. Cluster 6 discusses the effects of carbon emissions from urbanization and globalization in the Sub-Saharan region of Africa. As these studies are outside the mainstream of energy poverty research, they have not attracted much attention from scholars.

## 4. Conclusions and Future Research Directions

### 4.1. Conclusions

We collected papers on energy poverty published in the last 20 years (1999–2019) from the WoS databases and performed a visual analysis of external and internal characteristics. The study yielded the following findings:

With regard to external characteristics, energy poverty research went through the stages of beginning, growing, and maturity from 1999 to 2019. The year 2003 is the foundation year of energy poverty research. A total of 982 research institutions in 80 regions conducted research in this field. There is extensive cooperation between the countries, and the UK, the USA, Australia, and Italy play the most active role in the cooperation network. Compared to cooperation between countries, the intensity of inter-institutional cooperation is much lower. There is no core that connects the entire institutional network, except that the University of Nottingham, the University of Cape Town, and the University of Toronto play the role of a bridge between different clusters. Among journals that have published papers on energy poverty, *Energy Policy* pays attention to energy poverty longer than any other journal, and *Renewable and Sustainable Energy Reviews* represents the highest level of energy poverty research. Researchers in the field of energy poverty have formed small-scale co-authoring networks. Sovacool ranks first among all authors in terms of influence and research output. Of course, more countries, institutions, journals, and authors have joined the research on energy poverty in recent years, which has brought more insights and knowledge into this field.

With regard to internal characteristics, the focus of research in the field of energy poverty has been constantly changing in the last 20 years, accompanied by a constant increase in keywords and the expansion of related disciplines. The processes of integration, regeneration, expansion, and extinction are constantly occurring among the keywords. By analyzing the citation path evolution, we found the two most representative citation paths: one path starts from the concerns of energy-poor groups and stops at an ethical discussion on energy poverty; the second path is based on the existing technological path, continuously developing coping policies, evaluation methods, and a conceptual framework for dealing with energy poverty. Furthermore, through coupling analysis, we discovered four focuses of energy poverty research: improvement of definition, improvement of evaluation methods, effects of coping policy, and energy justice.

### 4.2. Future Research Directions

Poverty is a challenge for a human society that strives to achieve sustainable development in the 21st century. Energy poverty is an important component of poverty in modern society. At present, energy production and consumption are highly globalized, and energy poverty has become a common problem faced by developed and developing countries [86]. From the beginning, academic research on energy poverty has always progressed ahead of poverty reduction policy. Reliable research results provide the necessary support for policy making. We invite researchers around the world to carry out more extensive cross-regional cooperation. At the end of this paper, we recommend the following promising directions for future energy poverty research.

#### 4.2.1. Energy Poverty in Developing Countries

At present, developed countries have formed a unified policy framework for energy poverty at the national and even regional level and have achieved good policy results. Most energy poverty research is related to developed countries or economies. However, in developing countries, people face more serious difficulties related to energy. Due to relevant resource constraints, it is difficult for developing countries to pay much attention to energy poverty. Meanwhile, due to the lack of a unified policy framework, coping policies in developing countries usually take the form of specific projects such as the Brightness Project and Off-grid Electrification [19,87]. Among existing studies, very few focus on energy poverty in developing countries, because the relevant data is difficult to obtain and the governments of developing countries do not take the problem seriously. What is the situation with energy poverty in developing countries? What are the characteristics of coping policies in developing countries? Are these policies effective? Scholars need to answer these practical questions in future research.

#### 4.2.2. Impacts of Energy Poverty on Vulnerable Groups

Energy poverty has different negative effects on different groups [58]. The vast majority of energy poverty studies actually ignore the living conditions and real needs of vulnerable groups. Although some studies have discussed the impact of energy poverty on the health of children, the elderly and women, the scope of these studies is often limited to specific countries, and their conclusions are not universally relevant. Therefore, it is necessary to conduct studies covering wider regions and multiple vulnerable groups.

#### 4.2.3. Root Causes of Energy Poverty

Energy poverty is caused by a multitude of factors. So far, most researchers have investigated micro factors such as low income, old houses, and energy demand. Few scholars have tracked the root causes of energy poverty at the macro level. Taking China as an example, after the founding of the People’s Republic of China, the government planned central heating areas according to the standard of the Soviet Union, drawing a boundary line along the Qinling Mountains and the Huaihe River. As a result, residents in southern China have very limited means of heating in the cold season, thus facing higher health and fire risks [88]. Exploring and reflecting on the root causes of large-scale energy poverty can help the government to correct the flaws in existing policies and more comprehensively consider the well-being of all citizens in future macro-planning.

#### 4.2.4. Consequences of Emission Reduction Policies

Currently, when the necessity of tackling climate change and environmental pollution has become a consensus of human society, all countries must seriously fulfill their obligations to reduce emissions. However, it should be noted that it is extremely difficult for a country to achieve a balance between energy security, energy welfare, and environmental protection. The “energy trilemma” presented by the World Energy Council is a concise summary of this situation. From a policy practice perspective, some emission reduction policies not only serve the purpose of controlling carbon emissions but also help to ensure that people can enjoy reliable and cheap energy services. However, many emission reduction policies impose high heating costs on residents, leading them into a state of energy poverty [89]. It is therefore necessary to discuss the loss of energy welfare caused by emission reduction policies in order to help the government assess the rationality of current emission reduction policies and avoid the undesirable policy consequences.

## Figures and Tables

**Table 1:** The top 20 most productive countries in the field of energy poverty.

Rank	Country	Publications	Percent	Citations	h-Index
1	UK	335	32.9	7750	46
2	USA	134	13.2	3528	34
3	Spain	79	7.8	1126	16
4	Australia	77	7.6	1033	20
5	Germany	50	4.9	681	20
6	China	41	4	541	12
7	South Africa	41	4	449	12
8	Greece	36	3.5	929	15
9	India	33	3.2	450	12
10	Italy	33	3.2	522	12
11	France	29	2.8	550	13
12	Austria	28	2.8	785	16
13	New Zealand	24	2.4	420	12
14	Ireland	22	2.2	968	11
15	Canada	21	2.1	436	12
16	Sweden	21	2.1	425	12
17	Japan	20	2	384	8
18	Netherlands	20	2	542	10
19	Nigeria	18	1.8	206	9
20	Denmark	16	1.6	655	10

**Table 2:** Top 20 productive research institutions in the field of energy poverty.

Rank	Institution	Publications	Percent	Citations	h-Index
1	Univ Manchester	28	2.8	817	13
2	Columbia Univ	22	2.2	579	11
3	Univ Leeds	19	1.9	447	12
4	Univ Sussex	19	1.9	639	11
5	Univ Otago	18	1.8	320	9
6	Univ Queensland	18	1.8	155	8
7	Univ Birmingham	16	1.6	679	9
8	Univ Cambridge	16	1.6	246	9
9	Univ Oxford	16	1.6	280	8
10	Int Inst Appl Syst Anal	15	1.5	526	12
11	Natl Univ Singapore	15	1.5	789	11
12	Univ Seville	14	1.4	153	8
13	Aarhus Univ	13	1.3	633	9
14	Cardiff Univ	13	1.3	242	9
15	Natl Tech Univ Athens	13	1.3	183	7
16	Vermont Law Sch	13	1.3	604	10
17	Sheffield Hallam Univ	12	1.2	373	7
18	Univ Edinburgh	12	1.2	165	5
19	Univ College London	11	1.1	403	8
20	Univ Sheffield	11	1.1	59	4

## Data Availability

Not applicable.
